# Osteonecrosis of the Jaws in Dogs in Previously Irradiated Fields: 13 Cases (1989–2014)

**DOI:** 10.3389/fvets.2015.00005

**Published:** 2015-04-01

**Authors:** Ana Nemec, Boaz Arzi, Katherine Hansen, Brian G. Murphy, Milinda J. Lommer, Santiago Peralta, Frank J. M. Verstraete

**Affiliations:** ^1^Clinic for Surgery and Small Animals, Veterinary Faculty, University of Ljubljana, Ljubljana, Slovenia; ^2^Department of Surgical and Radiological Sciences, School of Veterinary Medicine, University of California at Davis, Davis, CA, USA; ^3^Department of Pathology, Microbiology and Immunology, School of Veterinary Medicine, University of California at Davis, Davis, CA, USA; ^4^Aggie Animal Dental Center, Mill Valley, CA, USA; ^5^Department of Clinical Sciences, College of Veterinary Medicine, Cornell University, Ithaca, NY, USA

**Keywords:** dog, jaw osteonecrosis, oral tumors, osteoradionecrosis, radiotherapy

## Abstract

The aim of this report was to characterize osteonecrosis of the jaws (ONJ) in previously irradiated fields in dogs that underwent radiotherapy (RT) for oral tumors. Osteoradionecrosis of the jaw (ORNJ) was further defined as osteonecrosis in a previously irradiated field in the absence of a tumor. Thirteen dogs clinically diagnosed with 15 ONJ lesions were included in this retrospective case series. Medical records were reviewed for: breed, sex, weight, and age of the patient, tumor type, location in the oral cavity and size, location of the ONJ, time from RT to ONJ onset, known duration of the ONJ, and tumor presence. Where available, histological assessment of tissues obtained from the primary tumor, and tissues obtained from the ONJ lesion, was performed, and computed tomographic (CT) images and dental radiographs were reviewed. RT and other treatment details were also reviewed. Twelve dogs developed ONJ in the area of the previously irradiated tumor or the jaw closest to the irradiated mucosal tumor. Recurrence of neoplasia was evident at the time of ONJ diagnosis in five dogs. Time from RT start to ONJ onset varied from 2 to 44 months. In three cases, ORNJ developed after dental extractions in the irradiated field. Dental radiographs mostly revealed a moth-eaten pattern of bone loss, CT mostly revealed osteolysis, and histopathology was consistent with osteonecrosis. To conclude, development of ONJ/ORNJ following RT is a rare, but potentially fatal complication. Patients undergoing RT may benefit from a comprehensive oral and dental examination and treatment prior to RT.

## Introduction

Radiotherapy (RT) is an important treatment modality for oral tumors in humans (1) and dogs (2–4); however, the irradiated patient is susceptible to developing early and long-term complications due to radiation (1–5). Osteoradionecrosis (ORN) of the jaw (ORNJ) is a devastating long-term complication of RT in humans that leads to reduced quality of life due to pain, dysphagia, facial deformation, and may even result in death (1, 6–8). Several theories about ORNJ pathogenesis have been suggested, with the latest considering bone damage being caused by radiation-induced fibrosis due to acute inflammation, production of free radicals, and chronic activation of fibroblasts (1). The reported incidence of ORNJ in humans ranges from 2 to 8% with a reported decline in recent years (1, 6–8). Late complications of RT in dogs are also rare (2), although the reported frequency of ORN is variable. In appendicular sites, ORN was reported in 4% of cases treated with orthovoltage RT in one study (9). However, Théon et al. (3, 4) reported that 6.4–7.6% of cases treated with megavoltage RT for canine oral tumors experienced ORNJ. Although orthovoltage is rarely used in current veterinary RT, Thrall (10) reported that 5% of dogs developed ORNJ after orthovoltage RT for canine acanthomatous ameloblastoma (CAA).

The diagnosis of ORNJ in humans is based primarily on clinical signs; usually ulceration of the mucosa with exposure of necrotic bone is noted. However, additional definitions and classifications have been employed, mainly depending on the persistence or recurrence of a primary tumor, radiologic signs, duration of bone exposure, and extent of the disease (1, 6, 11–13). Several factors predisposing a patient for ORNJ development have also been described in human medicine (1, 6, 7, 14), but reports are rare in veterinary medicine.

The aim of this retrospective study was to characterize osteonecrosis of the jaws (ONJ) in previously irradiated fields in a series of dogs that underwent RT for oral tumors as this entity is poorly defined in the veterinary literature. ORN of the jaw (ORNJ) was specifically defined for the purpose of this study as osteonecrosis in a previously irradiated field in the absence of persistent or recurrent tumor.

## Materials and Methods

### Selection criteria

Medical records from the small animal clinic of the William R. Pritchard Veterinary Medical Teaching Hospital of the University of California Davis, were searched for dogs that had diagnostic and/or treatment procedures for clinically diagnosed ONJ based upon clinical signs of ulceration of the mucous membrane with exposure of necrotic bone, regardless of tumor presence or recurrence. Ten cases met the inclusion criteria and were included in this study. Two additional cases were evaluated at Aggie Animal Dental Center, Mill Valley, CA, USA, and one additional case at the Department of Clinical Sciences, College of Veterinary Medicine, Cornell University, resulting in a total of 13 cases included in this study.

This investigation did not seek to identify the incidence of ONJ/ORNJ lesions in previously irradiated fields. The cases series reported here represent patients with ONJ/ORNJ who were seen at three different veterinary dentistry and oral surgery referral hospitals, regardless of where in the USA the cases were treated with RT.

### Medical records review

Medical records were reviewed, and the following data were recorded: breed, sex, weight, age of the patient at the start of RT, tumor type, location in the oral cavity and size (*T*) (15), location of the ONJ, time from RT to ONJ onset, and known duration of the ONJ. Time from RT to ONJ onset was determined as the time interval between the start of the (first) RT fraction and the onset of clinical signs typical of ONJ (ulceration of the mucosa with exposure of necrotic bone). Additionally, tumor presence or recurrence was recorded, and lesions where none was reported were considered “true” ORNJ (1). Known duration of the ONJ (i.e., duration of bone exposure) was determined as the interval between lesion identification (either by the client, referring veterinarian, or at the referral hospital) to the time of healing following surgical intervention, death, or last available follow-up. In cases, where the histology slides were available for review, histological assessment of the primary tumor before RT and of the tissues obtained from the site of the clinically determined ONJ, was performed. Additionally, computed tomographic (CT) images, if available at initial presentation (before RT) and at ONJ clinical diagnosis, were evaluated on a medical grade flat-screen monitor with commercially available software (eFilm Work station 3.4, eFilm Medical Inc., Toronto, ON, Canada). Dental radiographs were also reviewed. Similarly, RT details were also reviewed for information on radiation planning and delivery, and any additional treatments before and/or during the RT were also recorded.

## Results

### Population

Over a 25-year period (1989–2014), 13 dogs were clinically diagnosed with 15 ONJ lesions in previously irradiated fields (Table 1). Six dogs were mixed-breed, two were labrador retrievers, and one each of the following breeds: golden retriever, beagle, bloodhound, bichon frise, and brittany spaniel. There were five spayed females and eight castrated males. Dogs ranged in weight from 12.6 to 40 kg (median 25.5 kg, mean 25.4 kg). Dogs were 8–17 years old (median 11 years, mean 10.8 years) at the start of RT.

**Table 1:** **General characteristics of the primary tumor and osteonecrosis lesion**.

Case	Tumor location	Tumor type	T stage	Gross tumor disease at RT start	Gross tumor disease at RT end	Lesion location	Imaging of the lesion CT/dental radiographs	Histopathology	Time to lesion onset	Known lesion duration	Tumor recurrence at lesion onset	Outcome
1	L caudal maxilla (P3–P4)	SCC	T1	No	No	L caudal maxilla	No/yes	No	15.5 months, post-extractions	1 month	No	Lost to follow-up

2	R buccal mucosa	MM	N/A	Yes	No	R caudal maxilla	No/no	Yes – soft tissues only	3.5 months, post-extractions	3 months	No	Lost to follow-up

3	L caudal mandible (M1, lingual)	CAA	T1	Yes	No	R caudal mandible	Yes/yes	Yes	17 months	3 months	No	Remission after mandibular rim excision

4	L caudal maxilla	CAA	T3b	Yes	No	L caudal maxilla	Yes/yes – at the time of ONJ revision	Yes – soft tissues only	4 months	16 months	No	Stable disease after several débridements

5	Hard-soft palate junction	MM	T2	No	No	L caudal maxilla; later R caudal maxilla	Yes/yes; yes/yes	Yes; yes – soft tissues only	L side: 26 months (9 months post-extractions); R side: 44 months	L side: 2 months; R side: 1 month	No	L side: remission after 2 débridements; R side: remission after débridement

6	L caudal oral cavity/oro-pharynx	MM	T3a	Yes	No	L caudal mandible	No/yes	Yes – soft tissues only	6 months	1.5 months	No	Progressive lesion, euthanasia

7	Sublingual	MM	T1	Yes	No	L and R caudal mandibles	No/yes	Yes – not available for review	13 months	<1 month	No	Lost to follow-up

8	R caudal maxilla	SCC	T1	Yes	No	R caudal maxilla	No/yes	Yes – not available for review	3 months	2 months	No	Managed medically for 6 months prior to euthanasia

9	R caudal mandible	SCC	N/A	Yes	No	R caudal mandible	Yes/yes	No	2 months	1 month	Yes	Euthanasia suggested

10	L caudal maxilla (P3-M2)	CAA	T3b	Yes	Yes	L caudal maxilla	No/no	No	13 months	6 months	Yes	Slowly progressive lesion, euthanasia suggested

11	R caudal mandible (M1–M3, crossing midline)	SCC	T3b	Yes	No	R caudal mandible	No/no	No	6.5 months	1 month	Yes	Lost to follow-up

12	R caudal mandible (P3–M1)	CAA	T2	Yes	Yes	R caudal mandible	Yes/yes	Yes	11 months	3 months	Yes (SCC)	Remission after 2nd resection (R rostral mandibulectomy)

13	L caudal mandible	Undiffer-entiated sarcoma	T3a	Yes	Yes	L caudal mandible	Yes/yes	No	8.5 months	1 week	Yes	Euthanasia suggested

*L, left; R, right; P, premolar tooth; M, molar tooth; SCC, squamous cell carcinoma; MM, melanoma; CAA, canine acanthomatous ameloblastoma; T1, tumor smaller than 20 mm in maximum diameter; T2, tumor 20–40 mm in maximum diameter; T3, tumor larger than 40 mm maximum diameter (a, without bone involvement; b, with bone involvement)*.

### Characteristics of the primary tumor

The dogs were treated for each of CAA (*N* = 4), squamous cell carcinoma (SCC, *N* = 4), and melanoma (MM, *N* = 4), and for an undifferentiated sarcoma (*N* = 1). Slides of the primary neoplastic lesions were available for review in only three cases, with all other tumors diagnosed by outside commercial laboratories. In the three cases with slides available, the neoplasia diagnosis was confirmed histologically by a board-certified pathologist (BGM). Location and size of the oral tumors are presented in Table 1.

Pre-RT imaging (dental radiographs, skull radiographs, and/or CT) were available for six of the dogs (all T3 cases and one T2 case) and revealed bony invasion in three of the T3 cases. Data on the size of the tumors were not available for two dogs.

For radiation treatments, patients were treated using a Cobalt-60 (Co-60 unit) (El Dorado 8, AECL, Montreal, QC, Canada), 4 MV linear accelerator (Clinac 4, Varian, Palo Alto, CA, USA), or Clinac 2100 (Varian, Palo Alto, CA, USA). The source used for treatment of the Case 9 is unknown. Patients were treated with 32–54 Gy in 3–8 Gy fractions depending on the clinical radiation plan. The total number of fractions delivered ranged from 3 to 17 fractions. A total of four patients were treated with a palliative plan (4 × 8 Gy on a once- or twice-weekly basis) for gross CAA, MM, and SCC. A total of six patients were treated with a more coarsely fractionated definitive protocol (7 × 6 Gy once weekly or 12 × 4 Gy Monday–Wednesday–Friday) for CAA and MM. Three cases were treated with more typical fractionated definitive protocols (16 × 3 Gy or 18 × 3 Gy) for SCC and undifferentiated sarcoma. One patient received both a coarsely fractionated definitive protocol (12 × 4 Gy), followed by a palliative protocol 7 months later (Tables 2–4). Nine patients were treated with a hand-calculated plan, while four patients received a computer-based plan using 3D-conformal planning. Eleven patients had gross disease visible at the time of RT treatment, and three patients had gross disease noted in the record at the end of RT (Table 1). Both of the dogs without gross disease received definitive-intent radiation protocols, while those patients with gross disease received either palliative or definitive-intent protocols.

**Table 2:** **Reporting of radiation prescription and target doses as recommended by the American College of Veterinary Radiology-Radiation Oncology (ACVR-RO)**.

Patient number	RT intent	Dose per fraction (Gy)	Intended total dose (Gy)	Prescription point of dose	Target dose and dose variation within plan	Number of fractions	Time schedule
1	Definitive	3	48	1 cm	DMAX = 3.11 Gy, exit dose = 2.59 Gy; treat to 100% isodose line	16	Daily

2	Coarsely fractionated, definitive intent	6	42	3 cm	Skin dose = 6.9 Gy	7	Twice weekly

3	Palliative	8	32	3 cm	N/A	4	Weekly

4	Coarsely fractionated, definitive intent	4	48	5 cm	DMAX = 4.86 Gy, Skin dose = 3.99 Gy	12	MWF

5	Coarsely fractionated, definitive intent	4	48	PTV; normalize to 90%	MIN for PTV: 78.3%; MAX for PTV: 110.4%; mean for PTV: 95.7%;	12	MWF

6	Coarsely fractionated, definitive intent	4	48	depth 4 cm	DMAX = 4.58 Gy	12	MWF

7	Palliative	8	32	PTV; normalize to 96.4%	100% to isocenter; MIN for PTV: 85.9%; MAX for PTV: 103.5%; mean for PTV: 100.0%; modal 101.5%; median 100.8%	4	Weekly

8	Palliative	8	32	5 cm	Treat to 100% isodose line	4	Twice weekly

9	Definitive	3	54	PTV; 100% dose	DMAX = 3.23 Gy, isocenter dose = 3 Gy	18	Daily

10	Coarsely fractionated, definitive intent + Palliative 7 months after definitive	4 for definitive; unknown for follow-up palliative protocol	48 for definitive; unknown for follow-up palliative protocol	Record not available	Record not available	12 for definitive; 5 for palliative	MWF for definitive; twice weekly for palliative

11	Palliative	8	32	PTV; normalize to 90%	For delivered plan: MIN for PTV: 3.42 Gy; MAX for PTV: 18.2 Gy; Mean for PTV: 16.5 Gy; Range 3.24–18.2 Gy	3 delivered	Weekly

12	Coarsely fractionated, definitive intent	4	47	2.5 cm	Skin 3.4 Gy, dmax 4.24 Gy	12	MWF

13	Definitive	3	48	PTV, normalize to 88%	MIN for PTV: 29%; MAX for PTV: 117.2%; mean for PTV:109%	16	Daily

*MWF, Monday, Wednesday, Friday treatment days; GTV, gross tumor volume; CTV, clinical target volume; PTV, planning target volume; DMAX, depth of dose maximum; MIN, minimum dose; MAX, maximum dose*.

**Table 3:** **Reporting of radiation planning and target volumes as recommended by the ACVR-RO**.

Patient number	Plan type	TPS used	Heterogeneity correction	GTV	CTV	PTV
1	Manual	N/A	N/A	No gross tumor present	Not defined	Lmax P3–P4 + 3 cm

2	Manual	N/A	N/A	Pinpoint lesion	Not defined	Not defined

3	Manual	N/A	N/A	1 cm mass Lmand M1	Not defined	Not defined

4	Manual	N/A	N/A	L caudal maxilla, eroded L zygomatic bone, and into L nasal cavity	Not defined	Not defined

5	Computer	Eclipse	Yes	Not defined	Hard–soft palate junction, just L to the midline, going caudally to 1 cm proximal to the hyoid process	PTV = CTV + 4 mm Volume 9.52 cm^3^

6	Manual	Eclipse	N/A	Lmand M1–M3	Not defined	Lmand M1–M3 + 3 cm

7	Computer	Eclipse	Yes	Not defined	Not defined	volume 31.1 cm^3^

8	Manual	N/A	N/A	Not defined	Not defined	Not defined

9	Manual	N/A	N/A	Not defined	Not defined	Not defined

10	Manual	N/A	N/A	Record not available	Record not available	Record not available

11	Computer	Eclipse	No	Not defined	Rmand P4-ramus, with lateral and ventral ST to midline. 158.19 cm^3^	PTV = CTV + 3 mm, volume = 211.68 cm^3^

12	Manual	N/A	N/A	3 cm × 2 cm × 1.8 cm	Not defined	GTV + 3 cm

13	Computer	Eclipse	Yes	Not defined	Left caudal mandible, level of P1–M3, including ramus and tip of Max M1–M2, with surrounding lateral soft tissue and ventral soft tissue to midline; 66.96 cm^3^	PTV = CTV + 1 cm volume 192.33 m^3^

*Co-60, Cobalt-60 radiotherapy unit; CTV, clinical target volume; PTV, planning target volume; TPS, treatment planning system*.

**Table 4:** **Reporting of treatment delivery and beam information as recommended by the ACVR-RO**.

Patient number	Treatment interruptions	Deviation from protocol	Beam quality and energy	Equipment	SSD/SAD	Technique	Beam weighting	Field size (cm)	Beam modification and bolus	Skin bolus
1	No	No	6 MV	Clinac 2100	SSD	Single field	N/A	4.8 × 7.4	None	0.5 cm bolus

2	No	Reduced field size to 4 cm × 6 cm for doses #6–7	Co-60	Co-60	SSD	Single field	N/A	7 × 10 for 5 fractions, 4 × 6 for 2 fractions	Lead block in front of tongue. Gel agent inside cheek	None

3	No	No	4 MV	Clinac 4	SAD	Parallel opposed	Equal	5 × 8	None	None

4	No	No	Co-60	Co-60	SSD	Single field	N/A	6 × 8	Pink bolus in mouth and superflab between gingiva and lips	None

5	No	No	6MV	Clinac 2100	SAD	Parallel opposed	Equal	7.4 × 4.4	Mouth block; MLC on 270° beam	None

6	13-day break between fraction #7 and #8	Fraction #8 and #9 given at dose of 6 Gy after the 13-day break	4 MV	Clinac 4	SSD	Single field	N/A	5.5 × 9.5	Wet gauze in mouth	None

7	Not described	Not described	4 MV	Clinac 4	SAD	Parallel opposed	Equal	3.6 × 11.5	Wedges (15°) on both 0 and 180° beams	Not described

8	Not described	Not described	4 MV	Clinac 4	SAD	Parallel opposed	Equal	24 × 12	None	None

9	No	No	6 MV	Clinac 2100	SAD	Parallel opposed	Equal	8 × 16	None	1 cm bolus

10	Only received 5/6 prescribed palliative doses	Record not available	Co-60	Co-60	SSD	Record not available	Record not available	Record not available	Record not available	Record not available

11	Last treatment not given	Changed plan after 1st fraction	6 MV	Clinac 2100	SAD	Single field for first fraction, then parallel opposed	Equal	13.4 × 13.1; then 11.4 × 8.4	First fraction: MLC, Subsequent fractions: MLC on both fields, with wedge (45°, left) on 210; mouth block	1 cm on angle 210 field

12	No	First fraction received 3 Gy	4 MV	Clinac 4	SSD	Single field	N/A	6.5 × 4.5	None	0.5 cm bolus

13	No	2 treatments on same day (fraction 11–12)	6 MV	Clinac 2100	SAD	2 field: 90 and 275 beam angles	1.5 (beam angle 275), 0.5 (beam angle 90)	9 × 13.1 and 8.9 × 13.1	Mouth block; wedge 15°(right) on 90° beam	Wet gauze 0.5 cm

*MV, megavoltage; SSD, source to surface distance; SAD, source to axis distance; MLC, multi-leaf collimator*.

One patient had a treatment interruption of 13 days between fractions seven and eight, and the eighth fraction was given as a dose of 6 Gy; the reason is unknown. One patient received only five out of six planned doses. One patient received its first dose as a single field, after which the treatment was changed to a parallel-opposed two-field technique for subsequent fractions; the last treatment was not delivered due to poor patient condition. One patient received 3 Gy on its first definitive treatment day, but thereafter received 4 Gy fractions. The American College of Veterinary Radiology-Radiation Oncology recommended reporting data for radiation studies can be found in Tables 2–4 (16).

### Other treatments

Prior to RT, three dogs had their oral masses surgically reduced, including bone excision in one of these dogs. The other 10 dogs received no surgical intervention (other than biopsy) prior to RT. The stage of periodontal disease was not established in the majority of cases before RT, but five dogs received full periodontal treatment up to 3 months prior to initiation of RT.

Eleven dogs were treated with an antibiotic during or immediately after RT, but the timing and antibiotic selected varied. Only one dog received regular chlorhexidine gluconate oral rinses, starting in the middle of the RT when mucositis developed. In two dogs, a tea rinse was started at the time of mucositis development.

Three dogs received intratumoral chemotherapy (substance unknown), and five additional dogs received systemic chemotherapy, or a small molecule inhibitor (carboplatin, iniparib, or doxorubicin), or melanoma vaccine (Oncept, Merial) during RT or shortly after the RT was completed.

Seven dogs were treated with prednisone, with the treatment initiated during RT in six dogs and at the beginning of RT in one dog. The use of analgesics varied, but typically included an opioid and a non-steroidal anti-inflammatory drug in cases that were not treated with a steroid.

### Characteristics of the ONJ lesions

**Figure 1:**
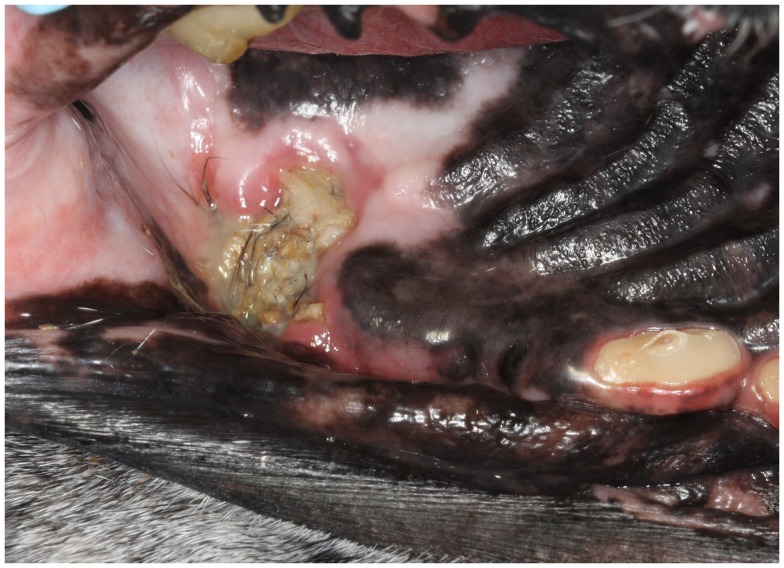
**An intraoral photograph of the dog in dorsal recumbency with ORNJ of the left maxilla (Case 5)**. There is an area of soft tissue necrosis at the level of missing left maxillary first molar tooth with underlying exposed necrotic bone covered partially by debris and hair. Note also the severe abrasion of all remaining teeth.

**Figure 2:**
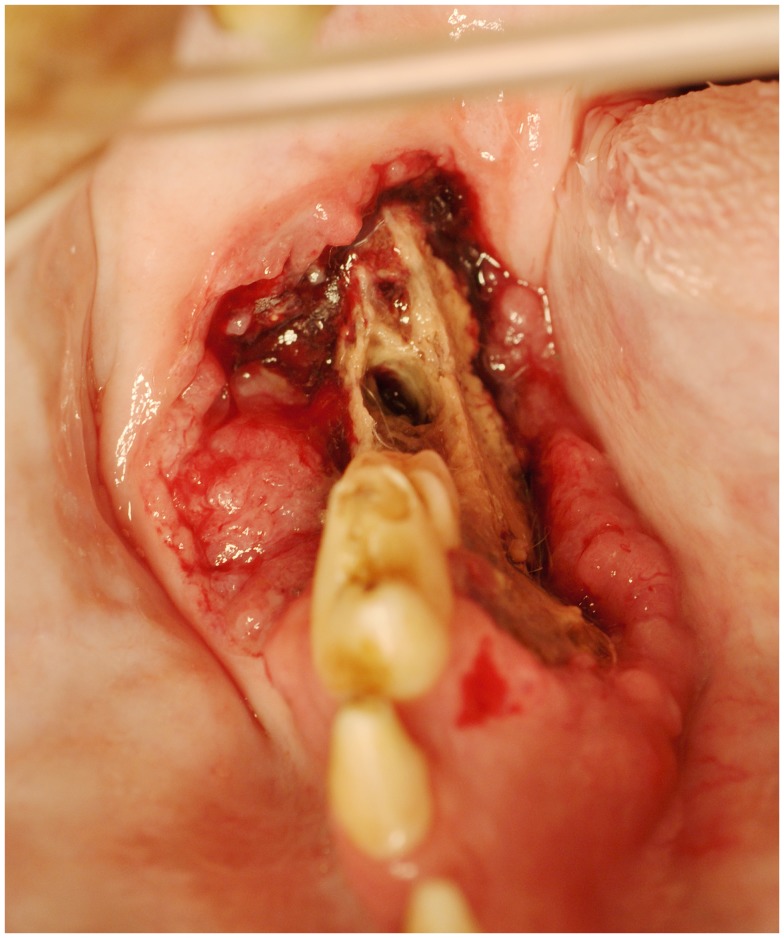
**An intraoral photograph of the dog in sternal recumbency with ONJ of the right mandible (Case 9)**. There is an extensive area of soft tissue necrosis with underlying exposed necrotic bone noted from the distal root of the right mandibular first molar tooth to the level of missing right mandibular third molar tooth. Note an uncomplicated crown fracture of the right mandibular first molar tooth. There is a soft tissue mass noticeable at the buccal aspect of the lesion, suggestive of neoplasia. Biopsy confirmed recurrent SCC.

Clinically, ONJ lesions were characterized as bone and soft tissue necrosis of variable extent (1) (Figure 1), regardless of evidence of tumor recurrence, and such lesions were detected in 13 cases. Recurrence of neoplasia (Figure 2) was evident on clinical and/or histopathological examination at the time of ONJ diagnosis in five dogs. All other cases were considered to be consistent with a diagnosis of ORNJ (Table 1).

As presented in Table 1, eight dogs developed lesions in the area of the previously irradiated tumor. Four dogs developed ORNJ lesion in the jaw adjacent to the irradiated tumor, and in one dog the ORNJ was found on the contralateral mandible. In one dog with a maxillary ORNJ lesion, an oronasal fistula (ONF) was noted at presentation, and in two additional dogs ONF developed after débridement. In two of these dogs, an orocutaneous fistula also developed.

Time from RT to ONJ onset varied from 2 to 44 months (median 9.8 months, mean 12.4 months), and for ORNJ specifically 3 to 44 months (median 13 months, mean 14.7 months) (Table 1). In one dog, ORNJ was noted 26 months after initiation of RT and 9 months after dental extractions were performed within the irradiation field; retained roots were later diagnosed. The same dog later developed ORNJ of the contralateral maxilla. Dental extractions were performed in the irradiated field in two other animals, with subsequent development of ORNJ.

Known duration of the ONJ varied from 1 week to 16 months (median 1.8 months, mean 2.9 months), and for ORNJ specifically from less than 1 to 16 months (median 2 months, mean 3.4 months) (Table 1).

**Figure 3:**
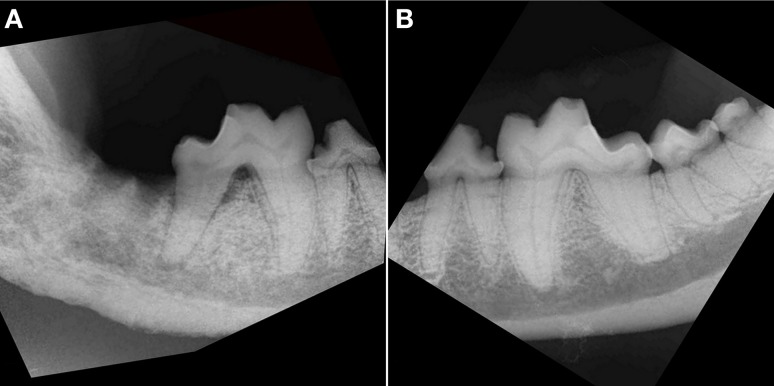
**Intraoral radiograph, lateral view of the right (A) and left (B) caudal mandible of the dog in Figure 2**. **(A)** There is an extensive area (all bone visible on the radiograph) of bony destruction, dominated by combined moth-eaten and permeative patterns of bone loss on the right mandible. Note also the generalized loss of definition of the mandibular canal, with a solid periosteal reaction, most prominent at the level of the distal root of right mandibular first molar tooth and missing right second and third molar teeth. There is horizontal and vertical bone loss at the distal root of the fractured right mandibular first molar tooth with furcation involvement. **(B)** Radiograph of the healthy left mandible shows minor horizontal bone loss at the mesial aspect of the left mandibular fourth premolar tooth and second molar tooth. There is a crown fracture of the left mandibular first molar tooth.

**Figure 4:**
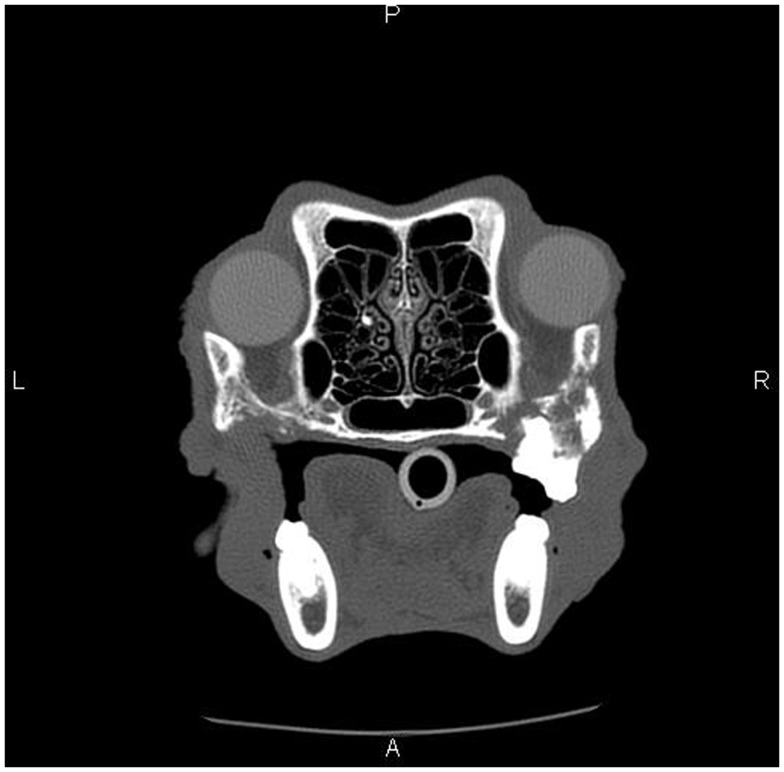
**Skull CT of the dog (Case 5) showing the section at the level of maxillary molar teeth**. There is extensive irregular osteolysis of the right caudal maxilla, palatine bone, and the rostral aspect of the zygomatic arch. There is remodeling of the caudal maxilla, palatine bone, and rostral zygomatic arch on the left side, where previous osteonecrosis was described (Figure 1). There is a missing left maxillary first molar tooth.

**Figure 5:**
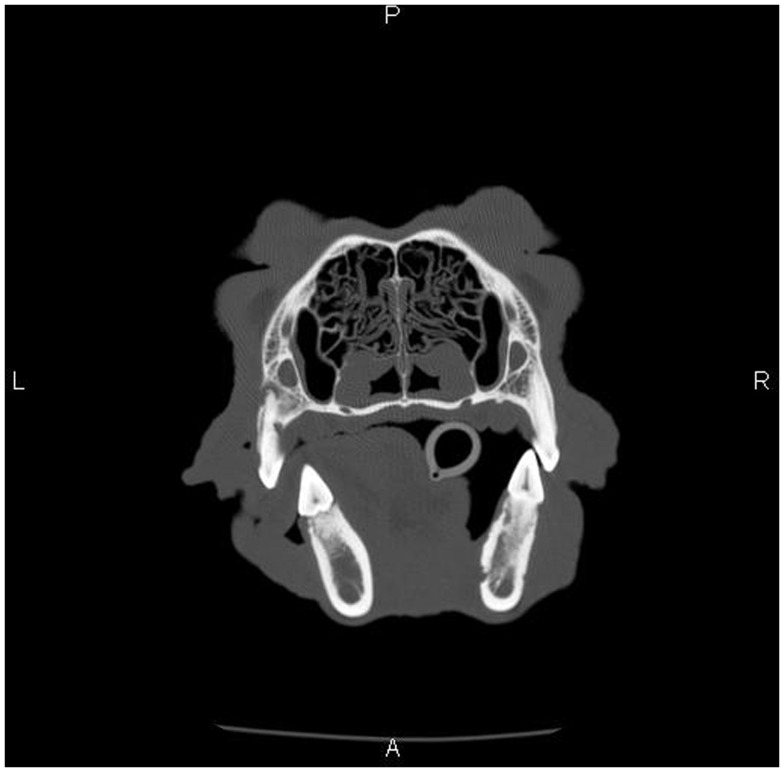
**Skull CT of the dog (Case 3) showing the section at the level of mandibular first molar teeth**. There is a diffuse thinning and multifocal pitting osteolysis of the cortical bone of the right mandibular body, and the changes are most pronounced in the medial cortex.

Dental radiographs from the time of ONJ diagnosis were available for 10 dogs (11 ONJ/ORNJ lesions). With the exception of one dog with bony changes in the area of the clinical lesion limited to horizontal bone loss associated with periodontitis, dental radiographs consistently revealed a moth-eaten pattern of bone loss (17). In six dogs, this moth-eaten bone loss was combined with a geographic and/or permeative pattern of bone loss (17). In three dogs where the mandible was involved, increased bone density with generalized loss of definition of the mandibular canal was diagnosed, with a solid periosteal reaction (17) noted in one dog (Figure 3). On CT, findings associated with the ONJ/ORNJ lesion were described as extensive and incomplete or irregular osteolysis (Figure 4) in five of six dogs, with one additional case demonstrating diffuse thinning of the mandible with multifocal pitting osteolysis (Figure 5).

**Figure 6:**
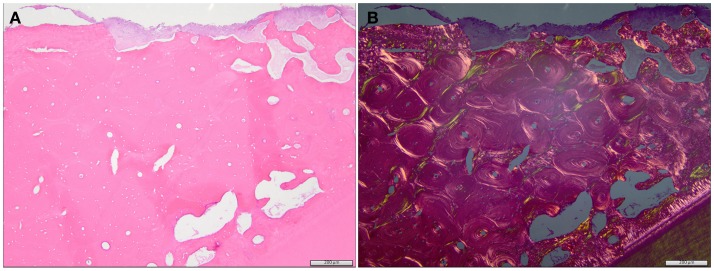
**Regular (A) and polarized (B) histological image of the specimen from the dog with an ORNJ lesion of the mandible (Case 3)**. **(A)** The alveolar bone comprises primarily lamellar, osteonal bone with limited interstitial woven bone. The bone is necrotic, as evidenced by diffuse, empty osteocyte lacunae, and attached basophilic to eosinophilic biofilm (top of image). Multifocal, irregularly spaced, and shaped resorption bays are present within the alveolar bone (clear spaces/osteoporosis). Tooth dentin and a thin rim of cementum are evident at the bottom of the image. These eosinophilic matrices are more evident in the polarized image **(B)**. A periodontal ligament is absent between the cementum and adjacent alveolar bone, consistent with ankylosis of the tooth.

Histopathology was performed in eight dogs with clinically diagnosed ONJ/ORNJ lesions, but specimens were only available for review in six dogs, including a dog where two biopsies of a progressive ONJ lesion were obtained 2 months apart. Bone and soft tissues were available for evaluation in three dogs, and only soft tissue was available for the other lesions. Soft tissue changes in all but one dog included pleocellular inflammation with mucosal hyperplasia and/or dysplasia and diffuse subepithelial fibrosis with multifocal vasculopathy and thrombosis. In one dog, the changes were most consistent with suppurative inflammation associated with an extensive area of necrotic tissue, and likely vascular thrombosis. Changes in the bone were described as locally extensive osteonecrosis with bone resorption and osteoporosis (Figure 6). Two of these lesions demonstrated features of osteomyelitis or had an attached biofilm on the exposed bone surface. In one dog, recurrent CAA was confirmed at the time of ONJ clinical diagnosis and was surgically removed with tumor-free margins (segmental mandibulectomy). However, 2 months later (13 months after the start of RT), the ONJ lesion was progressed and a right rostral mandibulectomy was performed. The histopathological evaluation of the right rostral mandible revealed osteonecrosis and SCC, which was removed with tumor-free and osteonecrosis-free margins. Review of the slides confirmed that primary lesion was CAA and the later SCC. Since the time for malignant transformation (5) was short, this may represent *de novo* tumor development.

In three dogs (including one dog with two ORNJ sites), the lesions healed after surgical intervention (one rim excision, one segmental and later unilateral rostral mandibulectomy, two débridement procedures). Two additional dogs received several débridement procedures to remove all grossly non-vital tissue to achieve stable or slowly progressive disease, or débridement was combined with hyperthermia. One patient was managed medically for 6 months prior to euthanasia. In one dog, progressive disease required another débridement procedure, but the dog was euthanized due to perioperative complications, and in another two dogs euthanasia was suggested due to the extent of the disease and poor quality of life. Four dogs were lost to follow-up.

## Discussion

The present study describes RT and associated treatments employed in a series of 13 dogs treated for odontogenic or non-odontogenic oral tumors. It further focuses on the characteristics of the ONJ/ORNJ lesions that later developed, especially the site of ONJ/ORNJ in relation to the primary tumor, time to onset, and duration of the necrotic lesion, imaging, and histopathology findings.

Radiotherapy alone can provide good tumor control in dogs depending on tumor size and type (2–4), and local control and survival time may be improved by combining RT with radiation sensitizers, surgery, or chemotherapy (2). Some studies suggest that chemotherapy use coincident with radiation may increase the risk of ORNJ development (1, 7). Conventional definitive RT in veterinary patients normally employs radiation fractions of 2–3.2 Gy once daily, 5 days a week, over 16–25 fractions (18). However, palliative RT protocols for veterinary patients have a wider variety of dosing schemes, with fraction sizes of 4–9 Gy given over a week or once weekly for 3–4 weeks being commonly used (19). RT does induce changes in healthy cells and tissues, and both deterministic and stochastic effects can occur (9). ORN is believed to be a deterministic effect, where below a certain threshold dose no effect is observed (9). In human patients, ORNJ is unlikely to occur if the radiation dose delivered by conventional RT is below 60 Gy (1, 6, 20). The risk of ORNJ in humans increases with the increased radiation dose (1, 6, 20). It is notable that the protocols used for RT in humans result in a much higher total dose to the tissue compared to veterinary protocols that normally do not exceed 60 Gy. Despite the lower total doses used in veterinary patients, ORN is seen in patients receiving doses of 32–48 Gy. The evident ORN at lower doses may be due to the higher dose per fraction (e.g., human patients often receive doses <2 Gy (7) for definitive protocols compared to veterinary patients that often receive 3–4 Gy for definitive therapy, resulting in a similarly high biologically effective dose despite the lower total dose delivered to dogs). Veterinary patients who have gross disease at the time of RT may also have tumor regression or tumor necrosis resulting in bone exposure, whereas patients with a healthy mucosal lining after surgery tend to have less bone exposure. This bone exposure could also contribute to bone lysis, and infection. In human studies, Co-60 RT (6), high total doses, short regimens using higher doses per fraction, and large field sizes are all associated with an increased risk of ORNJ (1, 20). Notably, the use of megavoltage RT results in reduced frequency of ORNJ compared to lower-energy, orthovoltage therapy (9). There is also evidence that continuous hyperfractionated accelerated RT possesses less risk for ORNJ development, although these protocols are not easily adapted to veterinary patients due to anesthesia and time constraints, and deliver smaller fractions to a low total dose (1, 7, 20). Definitive RT (without surgery) and adjunctive RT pose similar risks of developing ORN in humans (7). In this study, corticosteroid use during RT was employed to reduce the RT-induced inflammation of the oral mucosa and skin in seven of the dogs in this study. In a review looking at possible risks and predisposing factors, corticosteroids were found to reduce the risk of ORNJ by 96% in humans (1).

Upon presentation, the dogs in the present study had lesions in the oral cavity that were clinically described as soft tissue necrosis of variable extent with underlying exposed necrotic bone, warranting a clinical diagnosis of ONJ or, specifically, ORNJ if no tumor was noted (1). Although dogs <7 years of age were found to have a significantly higher risk of ORN development at appendicular sites in one study (9), all dogs in the present study were >8 years old. RT may have been selected rather than surgery in these older dogs as a less invasive treatment option, particularly for advanced, caudally located tumors. In such cases, curative-intent surgical resection is difficult or impossible; however, prolonged progression free intervals following RT are reported in dogs with large odontogenic tumors (3). The size of the tumors and degree of bone invasion may also influence ORNJ development in humans (1), although our case series included both small (T1) and large (T2, T3) tumors with and without bone involvement.

All but one of the ONJ/ORNJ lesions were found on the site of tumor irradiation or in the jaw closest to the irradiated tumor. Most human patients also develop ORNJ ipsilateral to the tumor inside the radiation field. However, bilateral involvement or involvement of the contralateral jaw may occur (21, 22), as also noted in our cases. It is likely that the exit radiation dose contributes to ORNJ in cases that develop bilateral or contralateral ORNJ after receiving a single field of radiation. In cases receiving the common “parallel opposed” radiation technique, wherein radiation beams are delivered from each side of the body, it is likely that both the entrance and exit dose of the beams contribute to development of ipsilateral, contralateral, or bilateral ORNJ lesions. In humans, higher susceptibility of the mandible, and especially the molar region, for ORNJ development is reported (1, 6, 7). Our study had a similar pattern of caudal location, but site predilection cannot be concluded from the present study.

The time to ORNJ onset varied greatly among dogs (3–44 months after start of RT), which is similar (1–69 months) to observations for mandibular ORN in humans (6). In human, ORNJ typically develops during the first 4–36 months after RT; the risk of developing ORNJ, however, remains for life (1). Because dogs have a life span considerably shorter than that of humans, it is difficult to project how long the risk of developing ORNJ remains in the veterinary population.

None of the dogs in this study had undergone major surgery before RT. Importantly, surgery performed immediately before RT may increase the risk of ORN development in humans (7). In at least three cases, however, ORNJ started after dental extractions. Post-RT surgery, and especially dental extractions in the radiation field, is considered one of the most important predisposing factors for ORNJ development in humans (1, 6, 14, 23), although an incidence of only 2% per tooth extracted has been reported (14). Reports in veterinary medicine are scarce, but dogs with intranasal neoplasia that had surgery performed after accelerated RT were more likely to develop ORN (24). It has been suggested for human patients that comprehensive oral/dental examination and dental extractions of all unrestorable or periodontally affected teeth should be performed at least 7–10 days before RT, and ideally 3 weeks before RT to allow healing (1, 6–8, 23). However, protocols for pre-RT dental evaluation have not been well established (7), even though dental status of patients scheduled for RT treatment may be poor (8).

Periodontal disease is a very common dental disease in middle-aged to older dogs (25); however, routine oral and dental evaluation and dental treatment were not commonly performed in the dogs included in this study. Ideally, oral/dental evaluation and treatment should be performed prior to initiating RT. If dental treatment is required during or after RT, surgical trauma should be minimized and dogs receiving radiation to the oral cavity, nasal cavity, or neighboring tissues should be managed by experienced oral surgeons (23). Preoperative and postoperative use of topical antiseptics and systemic antibiotics have also been widely employed, but direct evidence that such medical treatment reduces the incidence of ORNJ is currently lacking (14, 23). Human studies suggest that oral hygiene should be maximized during RT with the use of topical antiseptic mouthwashes (23); these washes were rarely performed in the dogs reported in this study.

The duration of bone exposure (1) may be very difficult to determine in dogs because it may go unnoticed by the owner for extended periods of time. This issue has also been debated in the human literature, and some authors suggest that bone exposure should be present for at least 1–3 months to prevent over diagnosis of ORNJ (1, 12). With only a brief period of observation, a clinician may erroneously diagnose ORNJ in a patient with mucositis or mucosal radionecrosis with exposed but not necrotic underlying bone (1). However, human ORNJ diagnostics suggest that if radiographic changes in the bone are observed at the first visit, a diagnosis of ORNJ can be made regardless of the period of bone exposure (12).

Imaging, including dental radiography and CT, can be very useful to further investigate ORNJ lesions (1). Dental radiographs of the lesions in advanced stages, when over 30% of bone mineral content is lost, usually reveal a mixed radiopaque/radiolucent lesion, representing bone destruction (1) similar to the present study. However, plain radiographs usually underestimate the extent of damaged bone and do not correlate with the clinical status (1, 12). A clear line of demarcation between devitalized and vital bone may not be observed (1), which is in agreement with the present report. CT has similar limitations (1) with the earliest osseous imaging findings of ORNJ being cortical defects and trabecular disorganization, air pockets, fractures, and sequestra formation in severely advanced lesions (21, 26). Even with imaging, the diagnosis of osteonecrosis may still be difficult, and its definition has been debated in human literature (1). During surgery, bleeding bone can provide a helpful guide to defining the extent of the necrosis (1), but histopathology is the ultimate diagnostic tool to confirm osteonecrosis (1, 13, 27). Histopathological examination reveals changes typical of a hypovascular and hypocellular/fibrotic tissue (1, 13, 27), which have been observed in the present study. Importantly, histopathology is also needed to rule out infection/osteomyelitis. In humans, microorganisms are considered to play a minor role in the pathophysiology of ORNJ (1, 27), but they may cause a superimposed infection of ORNJ (21, 27). Also, histopathology is necessary to evaluate persistence or recurrence of the neoplasia. In the present study, five dogs demonstrated evidence of neoplasia, which may have contributed to the osteonecrosis (1, 9).

Due to the retrospective nature of the study, certain limitations exist. First, histopathological confirmation of osteonecrosis was not available for all dogs. However, of the seven dogs lacking histopathology review of the clinically diagnosed ONJ lesion, and with an additional three dogs lacking bone in the samples, imaging was available for six. In these cases, the imaging confirmed bony involvement in five cases, supporting the clinical diagnosis of osteonecrosis (12). In one dog where no bony involvement was seen radiographically, progressive disease was observed over 1.5 months without evidence of tumor recurrence, which was most consistent with ORNJ. Also, of the 13 dogs included in this study, 6 had demonstrated evidence of tumor persistence or recurrence, which likely contributed to the osteonecrosis.

### Clinical relevance

The clinical consequences of ORNJ are significant, and treatment may not always be possible due to the extent and severity of the lesion. Although ORNJ appears to be a rare complication, clients should be advised of possible development of ORNJ following RT. In cases that are surgically amenable, especially smaller tumors, surgery should be considered a first line treatment, as complete removal of oral tumors can result in cure (28) with minimal effect on function and appearance (29). In addition, with the development of new reconstructive techniques (30), function and cosmesis can often be preserved even with larger resections. Finally, until with further studies we better understand ORNJ development in animals, we suggest an oral exam and treatment to be performed prior to RT for an oral tumor in dogs, as is the standard practice in human medicine.

## Author Contributions

All authors substantially contributed to the conception of the work, acquisition, analysis, and interpretation of the data. Agreement was reached by all authors for all aspects of the work in ensuring that questions related to the accuracy and integrity of all parts of the work are appropriately investigated and resolved. Ana Nemec drafted the manuscript, which was critically revised for important intellectual content and the final version approved to be published by all authors.

## Conflict of Interest Statement

The authors declare that the research was conducted in the absence of any commercial or financial relationships that could be construed as a potential conflict of interest.
